# WNT-activated bone grafts repair osteonecrotic lesions in aged animals

**DOI:** 10.1038/s41598-017-14395-9

**Published:** 2017-10-27

**Authors:** B. Salmon, B. Liu, E. Shen, T. Chen, J. Li, M. Gillette, R. C. Ransom, M. Ezran, C. A. Johnson, A. B. Castillo, W. J. Shen, F. B. Kraemer, A. A. Smith, J. A. Helms

**Affiliations:** 1 Division of Plastic and Reconstructive Surgery, Department of Surgery, Stanford School of Medicine, Stanford, CA USA; 2 Paris Descartes University - Sorbonne Paris Cité, EA 2496 - Orofacial Pathologies, Imaging and Biotherapies Lab and Dental Medicine Department, Bretonneau Hospital, HUPNVS, AP-HP, Paris, France; 3 Department of Mechanical and Aerospace Engineering, New York University Polytechnic School of Engineering, Brooklyn, NY USA; 4 Division of Endocrinology, Gerontology and Metabolism, Stanford University School of Medicine, Veterans Affairs Palo Alto Health Care System, Palo Alto, CA USA; 5 Present Address: State Key Laboratory of Oral Diseases, West China Hospital of Stomatology, Sichuan University, Chengdu, China; 6 Present Address: Department of Plastic Surgery, University of Pittsburgh Medical Center, Pittsburgh, PA USA

## Abstract

The Wnt pathway is a new target in bone therapeutic space. WNT proteins are potent stem cell activators and pro-osteogenic agents. Here, we gained insights into the molecular and cellular mechanisms responsible for liposome-reconstituted recombinant human WNT3A protein (L-WNT3A) efficacy to treat osteonecrotic defects. Skeletal injuries were coupled with cryoablation to create non-healing osteonecrotic defects in the diaphysis of the murine long bones. To replicate clinical therapy, osteonecrotic defects were treated with autologous bone graft, which were simulated by using bone graft material from syngeneic ACTB-eGFP-expressing mice. Control osteonecrotic defects received autografts alone; test sites received autografts treated *ex vivo* with L-WNT3A. *In vivo* µCT monitored healing over time and immunohistochemistry were used to track the fate of donor cells and assess their capacity to repair osteonecrotic defects according to age and WNT activation status. Collectively, analyses demonstrated that cells from the autograft directly contributed to repair of an osteonecrotic lesion, but this contribution diminished as the age of the donor increased. Pre-treating autografts from aged animals with L-WNT3A restored osteogenic capacity to autografts back to levels observed in autografts from young animals. A WNT therapeutic approach may therefore have utility in the treatment of osteonecrosis, especially in aged patients.

## Introduction

Osteonecrosis is a painful, progressive disease that primarily affects load-bearing bones in which a region of bone becomes necrotic because of an interruption to its blood supply. The most common cause of osteonecrosis is trauma^1^; in addition a number of other etiologies and risk factors have been identified (reviewed in^2^). For example, osteonecrosis can occur secondary to radiation^3^, chemotherapy^4^, corticosteroid use^5^, and alcoholism. Idiopathic osteonecrotic defects also occur. These lesions predominantly affect the femoral condyle, the epiphyses, metaphyses, and diaphyses of bones, and patients are typically over 50 years of age^6^. In the absence of clinical intervention, ~50% of individuals with osteonecrotic defects will suffer collapse of the affected bone, even if the lesion is initially small and the patient is asymptomatic^7^. This percent increases dramatically if the lesion is larger and/or the disease is symptomatic^8^ (reviewed in^9^). Once the affected hard tissue has broken down there is no therapy other than removing the dead bone and, if sufficient bone remains, placing a metal implant. When osteonecrosis is diagnosed in its early stages, drilling into the affected bone (known as core decompression surgery^10^) is sometimes employed in an effort to stimulate blood flow^11^. Drilling is occasionally supplemented with growth factors^12^. Bone grafting and cell therapy has also been proposed as a treatment^13,14^, but in bilateral or widespread disease, this approach is unfeasible. Non-surgical approaches to treat osteonecrosis have met with limited success^9,15^. The incidence of osteonecrosis is on the rise because the disease is caused by the drugs used to treat bone loss^16^. There was some hope that a new class of osteoporosis medications might avoid the risk of osteonecrosis but in a recent clinical trial of Romosozumab the same problems have surfaced^17^. The fear of osteonecrosis has led many older patients to forgo these drugs, which by 2012 has resulted in a 50% reduction in their use^18^. A safe, effective therapy for treating osteonecrosis, especially in older patients, is therefore of the utmost importance.

WNT proteins are potent, pro-osteogenic signals that control human bone mass^19^ and when delivered locally, accelerate bone healing^20^ (reviewed in^21,22^). Wnt signaling promotes bone formation by activating the osteogenic transcription factor Runx2, and represses bone resorption via a RANKL-dependent mechanism^20,23^. As a consequence, a number of systemic therapeutics has been developed to increase bone formation via the Wnt pathway. We developed a WNT protein therapeutic approach that activates the Wnt pathway, up regulates genes associated with osteogenic commitment and when delivered to a skeletal injury, accelerates healing^24^. Our goal here was to test a WNT-based strategy for the treatment of an osteonecrotic defect.

Existing animal models of osteonecrosis recapitulate some of the clinical features of femoral head osteonecrosis in humans^25–28^ but most are conducted in species that are intractable to detailed molecular, cellular, and histomorphometric evaluations of the injury response as a function of time. A murine model would allow for the assessment of molecular and cellular analyses of the early stages of osteonecrosis. For example, mice- like humans^5^– reproducibly develop osteonecrosis in response to glucocorticoids and therefore have served as useful models in which to identify new biomarkers^29^ of osteonecrotic disease^30^.

We used a controlled skeletal injury in combination with cryoablation to create an osteonecrotic defect and an environment unfavorable to spontaneous bone healing in mice. The cryoablative osteonecrosis model has been rigorously assessed in sheep^26^, dog^31^, and emu^32^. When cryoablation is coupled with skeletal injury in other species, the result is a stereotypical, quantifiable osteonecrotic lesion^25,33^; we found the same to be true in mice. Like many other models, a cryoablation/injury combination does not capture all of the facets observed in human disease^34^ but here, its high fidelity and reproducibility made it ideal for the purposes of this pilot study.

Autologous bone grafts (autografts) are the only graft source that contains viable osteogenic stem and progenitor cells, and that displays osteogenic, osteoinductive, and osteoconductive properties^35^. Autografts are harvested as part of a surgical procedure where mineralized bone matrix along with marrow aspirate and connective tissue stroma are harvested. The chemical properties of an autograft are largely dependent upon the presence of growth factors in the material, including Wnt proteins^35^. For instance, of the 19 mammalian Wnt ligands, 16 are expressed in freshly harvested autografts^36^. In addition, components of the Wnt pathway (including 3 Wnt target genes Axin2, Lef1, Tcf4 and the intracellular mediator beta catenin) are expressed in freshly harvested autografts^24,36^. Namely, our objective was to understand the extent to which a local WNT stimulus could be combined with an autograft and used to accelerate healing of a skeletal defect that included a large amount of osteonecrotic bone.

Because aging is a risk factor for some forms of osteonecrosis^37^, we first tested the efficacy of autografts from young animals then the efficacy of the same autografts from aged animals, and finally evaluated whether pre-treatment with a WNT protein therapeutic improved the healing of osteonecrotic defects created in elderly animals.

## Results

### A mouse model permits assessment of the early stage of osteonecrosis

**Figure 1:**
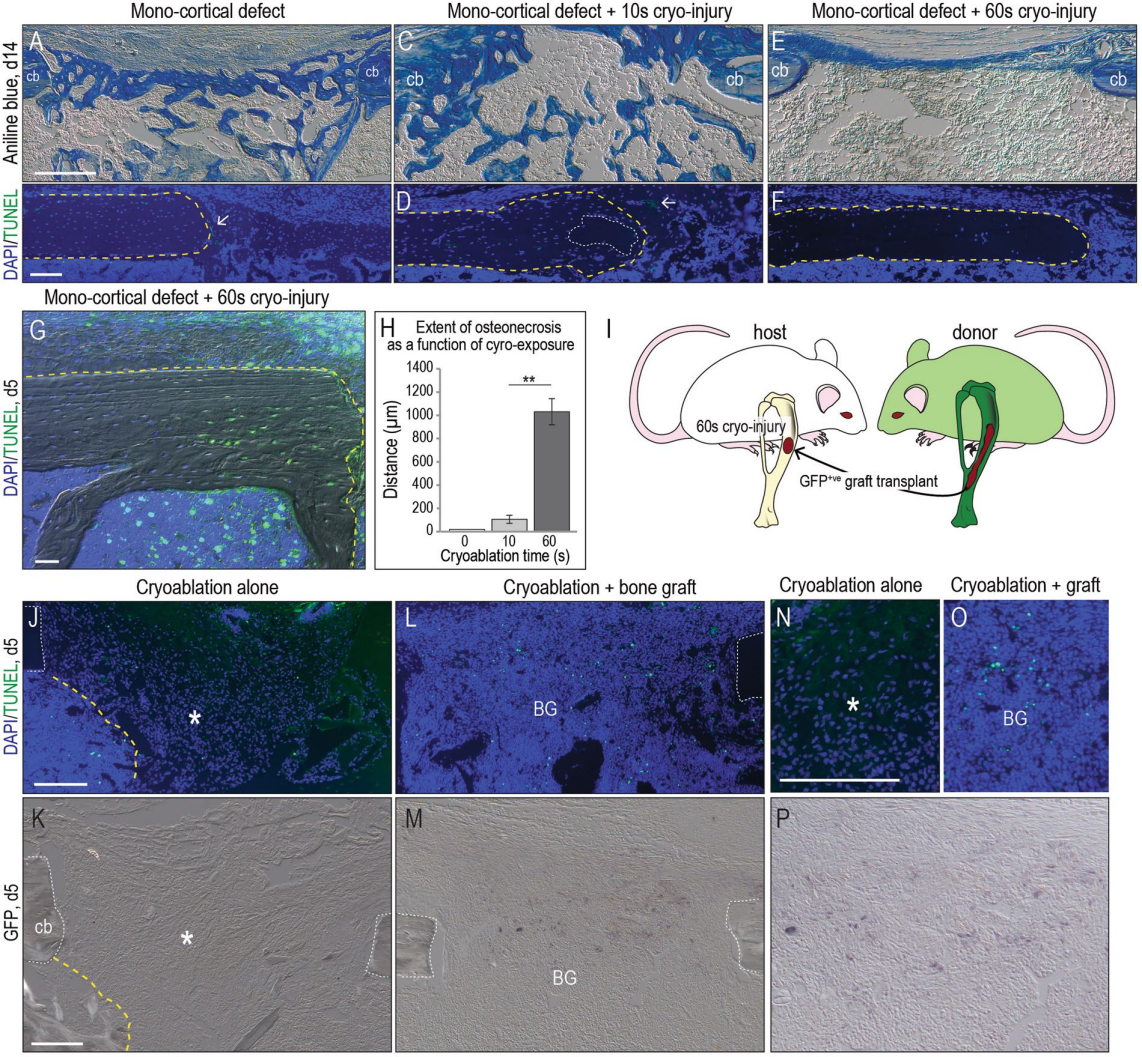
Modeling a mid-diaphyseal osteonecrotic defect and its management with an autograft. (**A**) Representative longitudinal tissue sections stained with Aniline blue, focusing on the mono-cortical defect site (N > 25); by post-surgery day 14 new mineralized tissue bridges the defect. (**B**) Adjacent tissue section stained with DAPI (blue) identifies viable cell nuclei in the cortical edge (delineated with a dotted yellow line) and in the defect site. No apoptotic cells were detected by TUNEL staining (green) on post-surgery day 14. (**C**) Representative tissue sections from a defect site subjected to 10 sec cryoablation where new mineralized tissue bridges the defect. (**D**) DAPI/TUNEL staining identifies a small area of necrosis (dotted white line) and few apoptotic cells (arrow). (**E**) Representative Aniline blue-stained tissue sections from a defect site subjected to 60 sec cryoablation. (**F**) DAPI staining identifies few viable cells in the cortical bone subjected to 60 sec cryoablation. (**G**) DAPI/TUNEL staining on tissue sections from a defect subjected to 60 sec cryoablation and analyzed on on post-surgery day 5. (**H**) Quantification of the distribution of DAPI^-ve^ osteocytes (e.g., empty lacunae) as a function of distance from the margin of the cut cortical bone (see Methods). (**I**) Schematic of experimental design, where bone graft is harvested from a GFP^+ve^ donor then grafted into a syngeneic host, which has sustained a mono-cortical defect subjected to cryoablation for 60 sec. Representative tissue sections, analyzed by (**J**) DAPI/TUNEL and (**K**) GFP immunostaining to illustrate cell density in an untreated osteonecrotic defect and the absence of a GFP signal. (**L**) Representative tissue sections, analyzed by DAPI/TUNEL and (**M**) GFP immunostaining in osteonecrotic defects treated with a GFP^+ve^ autograft. (**N**) High magnification of DAPI^+ve^ cells in an osteonecrotic lesion compared to (**O**) the DAPI signal in a lesion receiving an autograft. (**P**) GFP immunostaining attests to the persistence of donor cells inside the osteonecrotic area on post-surgery day 5. Abbreviations: BG, bone graft; cb, cortical bone; Scale bars, 100 µm; same magnification was applied in panels A,C,E, in panels B,D,F, in panels J,K and in panels N–P.

Our objective was to develop a mammalian model of osteonecrosis in order to decipher the molecular and cellular events that impacted subsequent healing of an osteonecrotic lesion. A mono-cortical defect was first produced in a femur or a tibia (Supplemental Fig. 1A,B), then combined with cryoablation (see Table 1 for experimental groups). The mono-cortical defect by itself exhibited robust healing: for example, bony bridging of the defect was reproducibly observed by post-surgery day 14 (Fig. 1A and see^38^). Creating the defect itself, however, caused minimal osteocyte death as revealed by co-staining with DAPI (to identify viable cell nuclei) and TUNEL (to detect cells undergoing apoptosis; Fig. 1B and see higher magnification image, Supplemental Fig. 1C). Exposing the mono-cortical defect to 10 sec of dry ice, delivered via the top of a metal drill bit (Supplemental Fig. 1D,E), did not appreciably alter the extent of cell death, or the rate of healing. For example, by post-surgery day 14 bony bridging still occurred in the 10 sec cryoablation group (Fig. 1C). There were small regions where DAPI staining was not detected in osteocytes, suggesting zones of osteonecrosis (dotted white line, Fig. 1D and Supplemental Fig. 1F) and foci of apoptotic cells (arrow, Fig. 1D).

**Table 1:** Experimental groups.

Mono − cortical defect	Defect + cryoablation (10 sec)	Defect + cryoablation (60 sec)	Defect + cryoablation (60 sec) + Graft^young^	Defect + cryoablation (60 sec) + Graft^aged^	Defect + cryoablation (60 sec) + Graft^aged + ^L-WNT3A	qRT-PCR analyses
4	3	14	17	21	25	5

Combining the mono-cortical defect with 60 sec of cryoablation reproducibly inhibited the otherwise robust healing observed with this defect. In place of the bony bridge a thin, fibrous tissue band was found on post-surgery day 14 (Fig. 1E). Representative tissue sections stained for DAPI and TUNEL revealed that the majority of the osteocytes in the cortical bone were dead (dotted yellow line, Fig. 1F and Supplemental Fig. 1G).

Analyses at an earlier time point (e.g., day 5) revealed the basis for this non-healing: the cryoablative injury resulted in widespread apoptosis (Fig. 1G). Death was not cell-type specific: osteocytes, as well as cells in the periosteum and endosteum, underwent programmed cell death (Fig. 1G). Histomorphometric analyses demonstrated a tight, very significant correspondence between the duration of the cryogenic insult and the osteocyte “kill zone” (Fig. 1H). So although cryoablation is not the cause of naturally-occurring osteonecrosis the method nonetheless proved useful because it allowed us to obtain reproducible, focal, necrotic lesions in bone that impaired normal healing in a mammal amenable to genetic, molecular, and cellular analyses.

### Mimicking autologous bone grafting for osteonecrotic lesions

Bone grafting has been performed with some success for the treatment of pre-collapse osteonecrotic femoral head defects, but in humans the fate of the autologous bone graft must be inferred from magnetic resonance imaging studies^13,39–41^. Here, we were able to exploit syngeneic mice to establish the fate of autografts used to treat osteonecrotic defects.

ACTB-eGFP^+ve^ mice served as donors and syngeneic, GFP^-ve^ expressing mice served as hosts (Fig. 1I). Mono-cortical defects +60 sec of cryoablation were produced in host mice and the resulting osteonecrotic lesions were then treated with bone graft material from GFP-expressing donors (Fig. 1I). Defects were examined five days later.

The first, obvious difference was the extent of cellularity in the osteonecrotic defect site. In the control group, which received no bone graft, osteonecrotic defects were sparsely populated by DAPI^+ve^ cells (asterisk, Fig. 1J,N). This sparse cell density contrasted with the adjacent, densely packed marrow space (delineated by a dotted yellow line). Osteonecrotic lesions treated with autografts were much more cellular, similar to the adjacent marrow space with some apoptotic cells within the graft (Fig. 1L,O). GFP immunostaining confirmed that cells in the defect site were indeed derived from the transplanted autograft (compare Fig. 1K control with M,P). Thus, after transplantation into a murine osteonecrotic defect autografts initially survived, similar to what has been reported in a large animal (pig) model of osteonecrosis^42^). Likewise, our data demonstrate that after transplantation into an osteonecrotic defect an autograft improves cell density in the lesion, equivalent to the nearby marrow; a similar conclusion has been reached by other investigators using magnetic resonance imaging in humans^40^. However, in humans it has not been possible to follow the fate of the bone graft because of a progressive decline in the uptake of gadolinium-based contrast agents^40^. Therefore our next series of experiments in mice focused on the fate of the autograft used to treat osteonecrotic lesions.

### Mitotic activity in an osteonecrotic defect is improved by autografting

**Figure 2:**
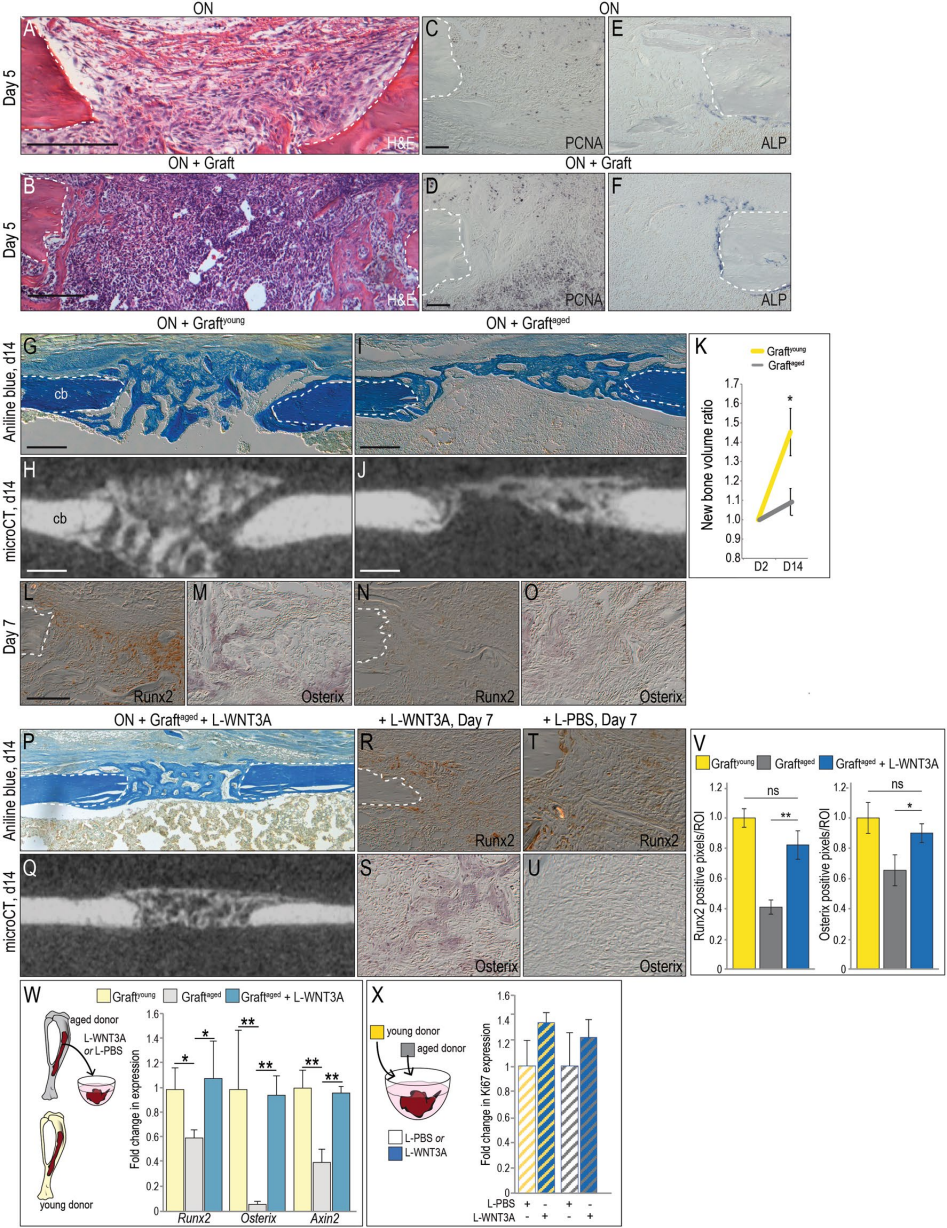
L-WNT3A restores the osteogenic potential of age-compromised bone graft. (**A**) Representative H&E stained longitudinal tissue sections, focusing on the osteonecrotic lesion and (**B**) the osteonecrotic defect with an autograft, on post-surgery day 5. (**C**) On adjacent tissue sections, PCNA immunostaining identifies mitotically active cells in the osteonecrotic defect and (**D**) in defects treated with autografts. (**E**) ALP activity indicates the state of mineralization on day 5 in osteonecrotic defects and (**F**) in defects treated with autografts. (**G**) A representative longitudinal tissue section through an osteonecrotic defect treated with bone graft from a young syngeneic donor (e.g., graft^young^) and harvested on post-surgery day 14; stained with Aniline blue to illustrate bony bridging. (**H**) Micro-CT image from the same sample with the same orientation, illustrating radiodense tissue in the defect. (**L**) Near-adjacent tissue sections immunostained for Runx2 and (**M**) Osterix on post-surgery day 7. (**I**) Tissue sections through an osteonecrotic defect treated with bone graft from an aged syngeneic donor (e.g., graft^aged^) on post-surgery day 14; Aniline blue staining shows incomplete bridging of the defect, verified by (**J**) Micro-CT. (**K**) Quantification of regenerated mineralized tissue in the defect site expressed as a ratio compared to the initial (post-surgery day 2) time point. (**N**,**O**) Tissue section through osteonecrotic defect treated with graft^aged^ and immunostained for (N) Runx2, and (O) Osterix on post-surgery day 7. (**P**) Aniline blue staining and (**Q**) Micro-CT imaging on post-surgery day 14 shows bony bridging of osteonecrotic defects that were transplanted with WNT-treated grafts^aged^. Runx2 and Osterix Immunohistochemical analyses of osteonecrotic defects treated with (**R**,**S**) graft^aged^ + (**T**,**U**) L-WNT3A compared to L-PBS. (**V**) Quantification of immunostaining resulting from L-WNT3A compared to L-PBS treated grafts. (**W**) Schematic of experimental design, where graft^aged^ was harvested from aged mice, treated with L-WNT3A (0.75 ng/µL) or L-PBS for 1 h then analyzed by qRT-PCR for relative expression of *Axin2*, *Osterix*, and *Runx2*. Expression levels of *Axin2*, *Osterix*, and *Runx2* in the samples are illustrated as fold-change relative to baseline expression in graft^young^ (yellow bars). (**X**) Schematic of experimental design where graft^aged^ and graft^young^ were treated with either L-PBS or L-WNT3A. Quantification of Ki67 expression is presented as fold-change relative to baseline in L-PBS-treated samples. Abbreviations: cb, cortical bone. Scale bars, 100 µm; same magnification was applied in panels C–F, in panels L–O,R-U and in panels G–J,P,Q. *p < 0.05.

Osteonecrotic lesions were examined using histology and immunostaining to assess the level of endogenous cell density and cell proliferation. On post-grafting day 5, H&E staining showed an obvious difference between defects that had received a bone graft versus those that had not (Fig. 2A,B). Compared to an osteonecrotic defect alone, those treated with grafts contained significantly more PCNA^+ve^ cells (Fig. 2C,D). Even at an early stage of the healing process osteonecrotic lesions treated with grafts also exhibited more ALP activity, indicating the initiation of extracellular matrix mineralization (Fig. 2E,F).

These data suggested that at least initially, autografting was beneficial. In some human patients, however, autografting of osteonecrotic lesions has proven to be ineffective and unpredictable^40,43,44^. Therefore we explored which features distinguished effective autografts from ineffective ones. We began by comparing the effect of patient age on graft success.

Autografting of an osteonecrotic defect led to bony bridging by post-transplant day 14, revealed by aniline blue histology and micro-CT imaging (Fig. 2G,H). These effective autografting cases were performed in young animals; when autografting was performed in aged (>12 months old) animals they proved to be far less effective, as shown by representative histologic evaluations and micro-CT imaging (Fig. 2I,J). Quantification of the ratio of new bone arising from graft^young^ vs. graft^aged^ was performed. These analyses demonstrated that even if the elderly donors were healthy, graft^aged^ samples were significantly less osteogenic (Fig. 2K). Expression of Runx2 and Osterix were below detectable limits in graft^aged^ as compared to their expression levels in graft^young^ (Fig. 2L–O).

### L-WNT3A amplifies the osteogenic potential of autografts from aged animals

A positive correlation exists between the state of endogenous Wnt responsiveness and the osteogenic potential of bone grafts^24,36^. Other groups have also demonstrated that amplified Wnt signaling causes an increase in bone mineral density and bone accrual^45–48^. Therefore we asked whether amplifying Wnt signaling in an autograft would be sufficient to rescue the age-related decline in osteogenic capacity we observed in graft^aged^ samples.

Bone grafts were collected from aged animals and incubated with either L-WNT3A or a liposomal formulation of PBS (L-PBS); the autografts were then used to treat the osteonecrotic defects as before. Bony bridging was evident by day 14 in the graft^aged^ + L-WNT3A treatment group but absent in the graft^aged^ + L-PBS group (compare Fig. 2P and I). Immunostaining demonstrated widespread expression of Runx2 and Osterix in WNT-treated autografts, which was absent in the PBS-treated grafts (compare Fig. 2R with T, and S with U). Quantification of the immunohistochemical signal was performed; relative to expression levels in graft^young^, Runx2 and Osterix were expressed at significantly lower levels in graft^aged^ (Fig. 2V). Treatment of graft^aged^ with L-WNT3A restored expression back to levels observed in graft^young^ (Fig. 2V).

These analyses raised an obvious question: was the L-WNT3A transforming the graft^aged^ into a more osteogenic material that healed the osteonecrotic defect? Or was the enhanced repair the result of a paracrine effect? To address this question graft^aged^ was harvested then treated for 1 h with L-WNT3A or L-PBS; immediately thereafter the levels of the Wnt target gene *Axin2* and *Runx2* and *Osterix* were quantified by RT-PCR. Relative to expression levels in graft^young^ (yellow bars), all three genes were significantly lower in L-PBS treated graft^aged^ (grey bars, Fig. 2W). Expression of all three genes in graft^aged^ rose in response to L-WNT3A, to the same levels observed in untreated graft^young^ (blue bars, Fig. 2W). Moreover, whether the bone graft material is harvested from a young donor or an aged donor, treatment with L-WNT3A increases the mitotic activity of the osteo-progenitor cells (Fig. 2X). Thus we conclude that L-WNT3A had a direct effect on the graft, by increasing expression of osteogenic proteins and inducing osteogenic differentiation of cells of the graft^aged^; together these effects result in the healing of an osteonecrotic lesion. We wondered if graft^young^ could be further improved by L-WNT3A treatment. Graft^young^ was treated with L-WNT3A or L-PBS as before then placed into an osteonecrotic defect. After 14 and 45 days no significant difference in autograft efficacy was detected between the two groups (Supplemental Fig. 2).

### L-WNT3A treated grafts from aged animals perform equivalent to bone grafts from young animals

**Figure 3:**
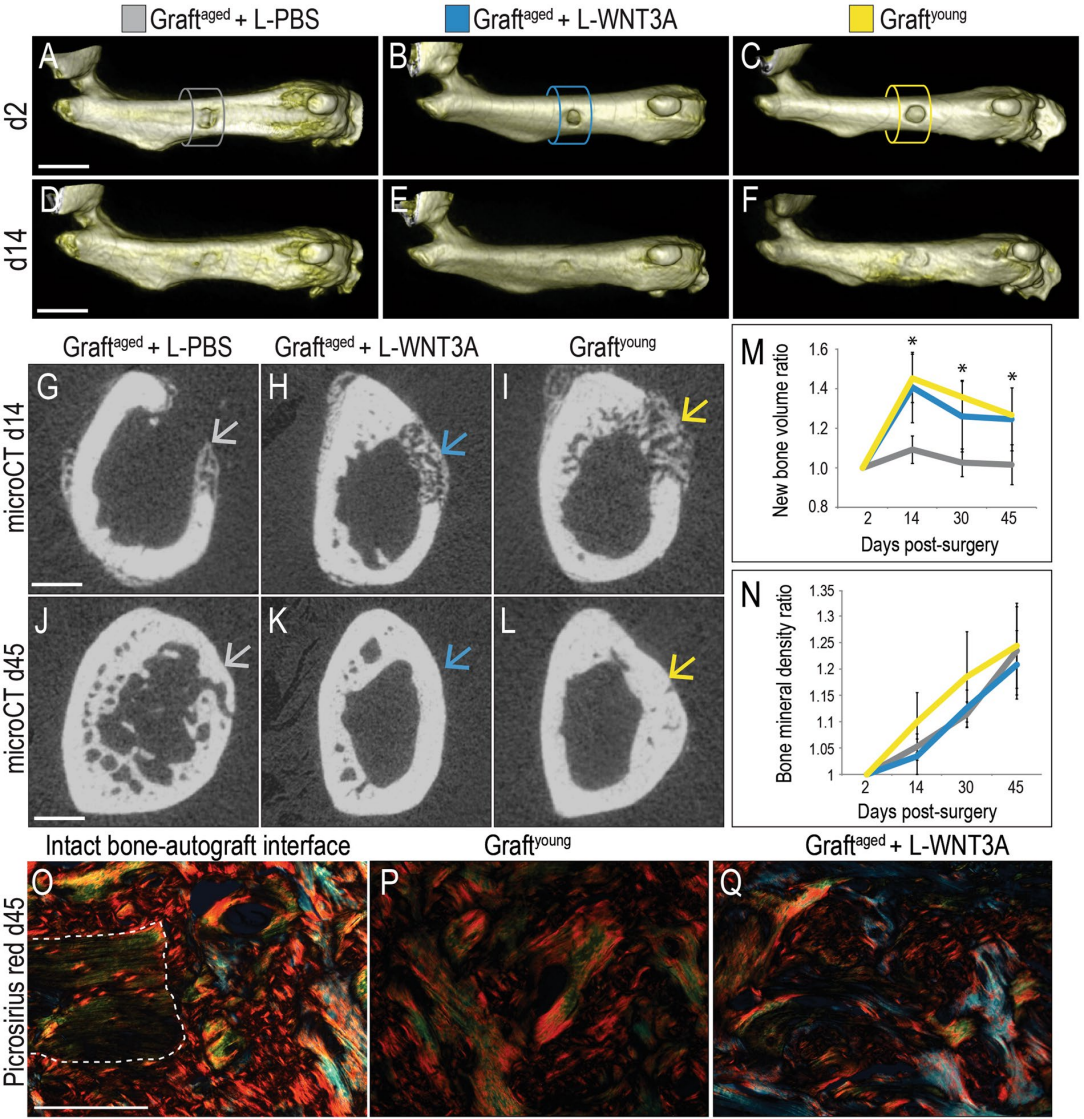
L-WNT3A improves the bone regeneration capacity of autografts from aged animals. Volume rendering of osteonecrotic defects treated with (**A**) graft^aged^ + L-PBS, (**B**) graft^aged^ + L-WNT3A and (**C**) graft^young^. (**D**–**F**) The same defects shown on post-surgery day 14. Axial micro-CT sections through the center of a representative osteonecrotic defects, treated with (**G**) graft^aged^ + L-PBS, (**H**) graft^aged^ + L-WNT3A and (**I**) graft^young^; arrows indicate healing sites. (**J**–**L**) The same defects shown on post-surgery day 45. (**M**) Quantification of bone volume in the defect. (**N**) Quantification of mineralized tissue density in the osteonecrotic defects, expressed as a ratio compared to the initial (post-surgery day 2) time point. (**O**) Picrosirius red staining, focusing on the interface between intact bone and the site of bone grafting showing the collagen organization for defects treated with (**P**) graft^young^ (**Q**) graft^aged^ + L-WNT3A. Scale bars, 2 mm in panels A–F (same magnification), 0.5 mm in panels G–L (same magnification) and 100 µm in panels O–Q (same magnification); Asterisk indicates statistical significance where p ≤ 0.05.

In all three treatments groups, micro-CT analyses showed that defects were of an equivalent size at the outset of the experiment (Fig. 3A–C; quantified in Fig. 3M). By post-operative day 14, cortical integrity was in the process of being restored in all three groups (Fig. 3D–F). Cross-sections revealed, however, a significant difference between the L-PBS treated graft^aged^ (Fig. 3G) and the L-WNT treated graft^aged^ (Fig. 3H). Both the L-WNT3A treated grafts and graft^young^ (Fig. 3I) generated significantly more new bone volume than the graft^aged^ treated with L-PBS (quantified in Fig. 3M). Likewise, the rate of new bone formation of L-WNT3A treated grafts was as fast as graft^young^, which significantly out-paced graft^aged^ treated with L-PBS (Fig. 3M). By post-grafting day 30 all defects showed evidence of bony bridging (Supplemental Fig. 3).

Between post-operative day 14 (Fig. 3G–I) and the conclusion of the experiment on day 45 (Fig. 3J–L) osteonecrotic defects treated with graft^young^ and graft^aged^ + L-WNT3A showed a gradual reduction in bone volume (Fig. 3M), commensurate with an increase in the relative density of mineralized tissue in the defects (Fig. 3N). Thus, there were no significant differences in bone mineral density between the groups; rather the difference was in bone volume.

On day 45, picrosirius red staining was used to visualize collagen organization in and around the defect. We first visualized the collagen organization where the graft was in direct contact with the intact bone (Fig. 3O). Here, picrosirius red staining illustrated a mature (red) collagen matrix (Fig. 3O). Defects treated with graft^young^ + L-PBS were filled with densely packed collagen extracellular matrix with a basket weave pattern (Fig. 3P). The mature bone resulting from WNT treatment appeared indistinguishable from healing bone resulting from graft^young^, with bone regenerated in response to graft^aged^ + L-WNT3A exhibiting a densely packed basket-weave pattern of collagen (Fig. 3Q).

**Figure 4:**
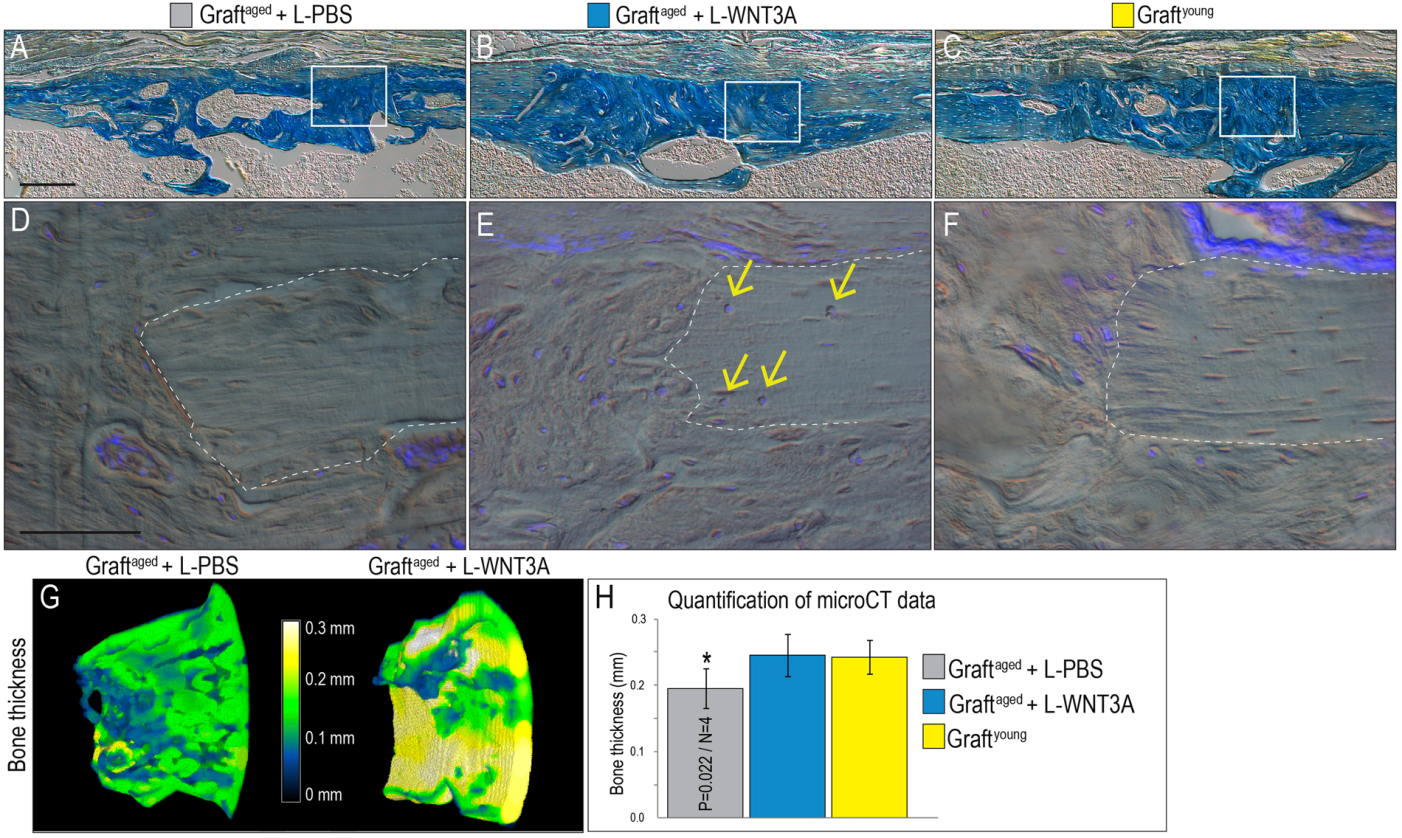
L-WNT3A treated autografts restore cortical integrity to osteonecrotic defects. Representative longitudinal tissue sections through osteonecrotic defects on post-surgery day 45; Aniline blue staining is used to illustrate the state of bone maturation after treatment with (**A**) graft^aged^ + L-PBS, (**B**) graft^aged^ + L-WNT3A and (**C**) graft^young^. DAPI staining for viable cells in the cortical edges of defects treated with (**D**) graft^aged^ + L-PBS, (**E**) graft^aged^ + L-WNT3A, and (**F**) graft^young^. (**G**) Color mapping of bone thickness obtained from micro-CT and measured in millimeters, at the treated defect sites on post-surgery day 45. Quantification of bone thickness in H. Scale bars, 100 µm; same magnification was applied in all panels A–C and in panels D–F.

We evaluated further the organization of the bony regenerate in our three treatment groups. In all cases, Aniline blue staining on representative tissue sections identified a lamellar osteoid matrix similar in organization to the adjacent, intact cortical bone (Fig. 4A–C), indicating that the microscopic structure of regenerated bone was similar to natural bone. Cortical lacunae remained devoid of DAP^I+ve^ signals in the graft^aged^ group (Fig. 4D) but viable cells were detected in and around the cortical border of the defect in the graft^aged^ + L-WNT3A and graft^young^ groups (Fig. 4E,F). Compared to graft^aged^ cases, those in the graft^aged^ + L-WNT3A treatment group generated significantly more bone in the osteonecrotic defect (Fig. 4G). When the overall thickness of the bony regenerate was analyzed, graft^aged^ + L-WNT3A and graft^young^ resulted in a significantly thicker bone (Fig. 4H). Thus, L-WNT3A treatment produced a significantly superior bony regenerate, equivalent to that generated by grafts from young animals.

## Discussion

### The challenges of developing an animal model for a disease without a well defined etiology

Currently there is no consensus on the etiology of osteonecrosis^15^, nor on the risk factors for the disease^34,49^. As a consequence, existing animal models are unlikely to fully recapitulate the human disease process. The cryoablative approach used here was based on previously published cryoablative models in dog^50^, sheep^26^, and emu^32^. Large animals are often employed for these studies because of similarities in the anatomy of the talar head and biochemical loading patterns^51–53^. Our goal here, however, was to characterize at a molecular and cellular level the early stages of osteonecrosis, and to develop a deeper understanding of the extent to which autografting may contribute to repair of the lesions. Because of these specific objectives we opted to develop an osteonecrotic defect model in an animal amenable to genetic, molecular, and cellular analyses. Although there are inherent difficulties in working with small animals, these limitations were offset by a number of distinct advantages. For example, there is a wide variety of transgenic mouse strains that recapitulate, in whole or in part, many human diseases, and being able to study the progression of osteonecrotic lesions in such animal models may reveal previously unappreciated relationships between a patient’s metabolic state and disease progression^54^. Another obvious advantage is sample size: studies in small animals permit the evaluation of multiple stages in a disease progression, and given the general lack of information about pre-collapse stages of osteonecrosis^55^, we considered this to be a significant benefit. The widespread availability of molecular and genetic assays, which are likely to yield critical insights into the etiology of osteonecrosis, was also a clear advantage.

Cryoablation has been extensively used to model osteonecrosis. It is an effective means to eliminate cells from the marginal bone and marrow cavity that would normally contribute to the healing process^56^, as well as to create volumetric lesions similar to those observed in humans^26^. Cryoablation, however, is not selective: cells in the vicinity of the thermal injury are killed whereas cells located at a distance are preserved. In that manner a cryoablative injury actually recapitulates one feature of osteonecrosis; namely, location-dependent, non-specific cell death^57^.

Both sheep and emu cryoablative models include a vascular ligation step, and the resulting vascular deficiency is thought to mimic one of the risk factors for osteonecrosis^9^. Our cryoablation model did not include an underlying vascular ligation step; although local blood supply was curtailed by the cryoablation step, there was no additional impedance in vascularization. This is a limitation of our study. Even without vascular compromise, 60 sec of cryoablation was sufficient to halt the normally robust program of bone healing typically observed in mice (Fig. 1).

### Autografting osteonecrotic defects is most effective in young animals

Current approaches for treating osteonecrotic defects are largely considered to be ineffective (reviewed in^58^). Pharmacological treatments require a firm understanding the pathoetiology of the disease but this is still lacking and consequently most of these approaches have not proven successful^59^. Osteonecrotic defects may benefit from autologous mesenchymal stem cell (MSC) treatment^60–63^ but other studies clearly demonstrate that these cell populations have diminished osteogenic differentiation capacity in patients with osteonecrotic defects^64^. This lack of success has led investigators to suggest that bone grafting procedures^13,65^ reviewed in^66^, coupled with biochemical agents, may be the best strategy for treating pre-collapse osteonecrosis^9^. Compared with existing orthopedic biomaterials including recombinant Bone Morphogenetic Protein 2 (BMP2), whose use has been linked to life-threatening adverse events including cancers^67,68^ and allogeneic cell products that have no clear mechanism of action^69,70^, a WNT protein therapeutic represents a potentially safer, more efficacious means to accelerate bone healing. Our approach avoids potential issues with acquired resistance because L-WNT3A treatment of a patient’s autograft results in enhanced Wnt signaling only in the autograft and not in surrounding tissues. With a reproducible model of osteonecrosis in hand, we were able to evaluate the contribution and potential efficacy of autologous bone grafting for osteonecrotic defect (Figs 1 and 2).

Autografting is unnecessarily traumatic for mice and can be effectively mimicked by using bone grafts harvested from syngeneic donors^71^. In our experiments bone grafts were harvested from mice constitutively expressing GFP so that cells from the graft could be identified by their GFP^+ve^ status. This allowed us to directly evaluate the extent to which autografts actually contributed to repair of osteonecrotic defects. We found that the quantity and quality of the bony regenerate was significantly decreased if the donors were elderly (Fig. 2). The same relationship holds true for humans^72^.

### Wnts and the decline in osteogenic capacity of autografts

We gained some insights into the basis for this age-related decline in the osteogenic efficacy of autografts from elderly animals. When the bone graft is transplanted into a skeletal defect, a critical first step is engraftment and survival^36^. Next, surviving cells must begin to express osteogenic proteins and then differentiate into osteoblasts, secrete a mineralized matrix, and eventually contribute to healing^24^. All of these steps occur when the graft is harvested from a young animal, and they all occur even when the graft is placed into an osteonecrotic defect (Figs 2 L,M and 4C,F). When the graft is harvested from an aged animal the cells also engrafted but endogenous osteogenic gene expression was significantly reduced (Fig. 2N,O). All subsequent steps, where cells differentiate into osteoblasts and secrete a mineralized matrix to heal the defect, are also reduced if the donor is elderly (Fig. 2T,U). The question then becomes, what is the cause for this age-related decline in osteogenic differentiation?

We have some clues. First, data acquired here and elsewhere indicate that it is a Wnt dependent event; as animals (including humans) age, the osteogenic capacity of an autograft deteriorates, along with its endogenous Wnt responsive status. We know this because following L-WNT3A treatment osteogenic protein expression levels in graft^aged^ return to those seen in graft^young^ (Fig. 2) and see^22,24^. Indirect evidence from human studies supports a role for Wnt signaling: when Wnt signaling is reduced, either because of elevated DKK1 or Sclerostin, the result is osteoporotic bone^73,74^. The age-related decline in osteogenic differentiation of an autograft may also be related to a deterioration in the number and/or function of stem/osteoprogenitor cells^75,76^. At least in animal studies treatment with L-WNT3A activates the stem/progenitor population in an autograft^36^, leading to their accelerated differentiation into osteoblasts^24^. Some data suggest that patients with osteonecrosis have fewer^77^ and/or less active^78^ mesenchymal stem cells compared to healthy control groups (reviewed in^63^). Also, it has been reported that mesenchymal stem cells from patients with osteonecrosis show less of an ability to differentiate into osteoblasts compared to analogous stem cell populations from patients with osteoarthritis^64^. Finally, Wnt signals are known to activate stem and osteoprogenitor cells^24^, both of which are contained within an autograft^24,36^. This latter point helps explain why L-WNT3A treatment restores osteogenic potential to autografts from aged animals (Fig. 4).

## Conclusion

A primary guiding principal in the field of regenerative medicine is “*restitutio ad integrum*”, i.e., restoration of an entity back to its original condition. Skeletal healing in young patients largely achieves this goal, but in humans and other mammals this capacity deteriorates with age. Precisely why osteonecrotic lesions in the young do not achieve *restitutio ad integrum* is not clear, however and this is at least part of the reason that the pathoetiology of osteonecrosis has come under intense scrutiny.

In order to simulate a non-healing status, very large osteonecrotic lesions were generated in mice and these failed to heal spontaneously. When considering the diameter of the murine tibia and the relative size of the defect we created (which was just under the critical limit whereby tibial integrity could be maintained), an equivalent osteonecrotic defect in humans would be on the order of 1.4 cm in diameter. Autografting and its contribution to healing the defect could be easily assessed in the mouse model and these studies provided the first clues into why osteonecrotic lesions typically fail to repair. The presence of proliferative osteoprogenitor cells in the grafted group directly compensated for the lack of healing seen in untreated osteonecrotic defects (Fig. 2). It was specifically the ability of the grafted cells to proliferate and then differentiate that was compromised when autografts were harvested from elderly donors^24^. The WNT stimulus, provided in the formulation of L-WNT3A, was sufficient to overcome the age-related deficit (Figs 2 and 3). In other disease states such as osteoporosis, elevation of Wnt signaling has thus far proven effective in increasing bone mineral density of the aged skeleton^79^. A strategy based on a similar molecular approach may therefore prove useful in treating osteonecrotic defects.

## Methods

### Animals, surgery, and the osteonecrotic defect model

All experiments were performed in accordance with Stanford animal healthcare guidelines and regulations. Beta-actin-enhanced green fluorescent protein (ACTB-eGFP) transgenic mice were purchased from Jackson laboratories (Bar Harbor, ME, USA) and crossed with littermates for >10 generations to obtain syngeneic ACTB-eGFP^+ve^ and GFP^-ve^ mice. For all experiments, 3–4 month-old mice were considered young and mice ≥12 months were considered aged. In bone grafting experiments, ACTB-eGFP^+ve^ mice served as donors and syngeneic GFP^-ve^ mice served as hosts. All mice were maintained under a 12-hour light/dark cycle with access to food and water ad libitum. The numbers of animals in each experimental group are shown in Table 1.

After ketamine/xylazine anesthesia, a full thickness flap was created on the anterior surface of the tibia or the femur diaphysis and a 1.0 mm mono-cortical defect was generated using a dental drill. In a subset of animals, cryoablation was performed immediately afterwards and was achieved via thermal transfer^25,26^, where the top of a metal drill bit was put in contact with a piece of dry ice (surface temperature of −78.5 °C) for either 10 sec or 60 sec (e.g., the defect + cryoablation groups; See Supplemental Fig. 1).

### Autologous bone grafting

Bone graft material was harvested from ACTB-eGFP^+ve^ mice donors and transplanted into syngeneic eGFP^-ve^ hosts, thereby simulating an autograft^36^. The recipients were matched in age with the donors. In brief, ACTB-eGFP^+ve^ donor mice were euthanized for other experimental purposes, femurs and tibiae were collected and split lengthwise, and bone graft was collected by gently scraping the endosteal surface of the marrow cavity with a dental excavator. In experiments where the bone graft was analyzed for mitotic activity, the cells were separated from bone chips using gentle centrifugation (e.g., 14 g for 2 min).

### *Ex vivo* treatment of bone grafts with liposome-reconstituted human WNT3A protein

Liposome-reconstituted human WNT3A protein (L-WNT3A; 10 µL) at a concentration of 0.75 ng/µL was combined with a solution containing Dulbecco’s Modified Eagle’s Medium (DMEM). A control solution contained an identical formulation of liposomal phosphate buffered saline (L-PBS) in DMEM. Immediately after harvest, the bone graft was incubated in one of the two solutions *ex vivo* for 1 h at 23 °C. Thereafter the bone graft was removed from the solution and transplanted into an osteonecrotic defect. Muscle and skin were closed, and mice received subcutaneous injections of buprenorphine (0.1 mg/kg) for analgesia.

### Micro-Computed tomography (CT) imaging

Scanning and analyses followed published guidelines^80^. *In vivo* three-dimensional micro-CT imaging was performed at various times after surgery. A µCT tomography data-acquisition system (RS150/Microview; GE Healthcare, Amersham, UK) at 49 μm voxel size (70 kV, 50 mA, 720 views, 20 ms) was used for scanning, calibration (Hounsfield units), reconstruction and image analyses. Bone volume and density were evaluated in a same region of interest (ROI) indicated with a cylinder (e.g., Fig. 3A–C) where the ROI was centered on the defect and extended for 2.5 mm in longitudinal axis. Regarding the variability of the anatomical location of the defect, initial scans performed on post-operative day 2 were used as references to assess the newly formed bone for each sample. In addition, *ex vivo* high-resolution acquisitions (VivaCT 40, Scanco, Brüttisellen, Switzerland) at 10.5 μm voxel size (55 kV, 145 μA, 347 ms integration time), were performed at post-operative days 14 and 45. Multiplanar reconstruction and volume rendering were carried out using Osirix software (version 5.8, Pixmeo, Bernex, Switzerland). Results were presented in the form of mean ± standard deviation, with N equal to the number of samples analyzed. Differences between data sets were determined using a Student T-test in XLStat software version (Addinsoft, Paris, France). A p-value < 0.05 was considered statistically significant.

### Tissue staining, immunohistochemistry (IHC)

Tibiae/femurs were harvested at the specified time points and fixed in 4% paraformaldehyde (PFA) at 4 °C for 12 hours. Samples were decalcified in 19% EDTA, dehydrated in a graded ethanol series, embedded in paraffin, and sectioned at 8 µm thickness. Antigen retrieval was performed by incubating slides with IHC-Tek Epitope Retrieval Solution (IHC WORLD, Woodstock, MD) at 95 °C for 20 min. Endogenous peroxidase activity was quenched by 3% hydrogen peroxide. Slides were blocked with 10% goat serum and incubated with primary antibodies. Antibodies include rabbit polyclonal anti-GFP (2956S, Cell Signaling Technology Inc., Danvers, MA), rabbit polyclonal anti-PCNA (Ab18197, Abcam, Cambridge, MA), rabbit polyclonal anti-Osterix (Ab22552, Abcam), rabbit polyclonal anti-RunX2 (Ab23981, abcam) and rabbit polyclonal anti-Ki67 (RB-9043 ThermoFischer Scientific). Anti-rabbit biotinylated secondary antibodies were used (Vector Laboratories, Burlingame, CA) and signals were detected using DAB (Vector Laboratories). Hemotoxylin and Eosin (H&E), Aniline blue, picrosirius red, and ALP staining were performed as described^20^. DAPI/TUNEL (Roche) assays were performed as previously described^24^). The quantification of the Ki67 signal was used to evaluate cell proliferation in the bone graft samples as previously described^81^.To quantify the extent of cell death extension induced by cryoablation the distance between the edge of the defect and the viable cortical zone (e.g., DAPI^-ve^ osteocytes limit) was evaluated using ImageJ software (1.50b).

### Quantitative Reverse Transcription-Polymerase Chain Reaction (qRT-PCR)

Bone graft was collected as described then incubated in 200 μL of DMEM supplemented with 10% fetal bovine serum (FBS) containing L-PBS or L-WNT3A (0.75 ng/µL). After 1 h incubation at 23 °C, 1 ml fresh DMEM was added, samples were incubated at 37 °C, 5% CO_2_ for an additional 23 h, washed with PBS, and prepared for RNA extraction. Following homogenization in Trizol solution RNA was isolated with RNeasy plus mini kit (Qiagen, Valencia, CA). Reverse transcription was performed with SuperScript III First-Strand Synthesis SuperMix for qRT-PCR Kit (Life Technologies, Grand Island, NY) as described^24^. Primers sequences (5′ to 3′) are as following: ß-actin, [for-GGAATGGGTCAGAAGGACTC], [rev-CATGTCGTCCCAGTTGGTAA]; Osterix, [for GGAGACCTTGCTCGTAGAT TTC], [rev- GGGATCTTAGTGACTGCCTAAC]; Runx2, [for- TGGCTTGGGTTTC AGGTTAG], [rev- CCTCCCTTCTCAACCTCTAATG]; Axin2, [for-TCATTTTCC GAGAACCCACCGC], [rev- GCTCCAGTTTCAGTTTCTCCAGCC]. Results were normalized to expression levels of ß-actin.

### Ethics

The Stanford Committee on Animal Research approved all procedures.

### Data availability

The datasets generated during and/or analysed during the current study are available from the corresponding author on reasonable request.

## Electronic supplementary material

Supplementary information
