# Author Correction: WNT-activated bone grafts repair osteonecrotic lesions in aged animals

**DOI:** 10.1038/s41598-018-24601-x

**Published:** 2018-04-17

**Authors:** B. Salmon, B. Liu, E. Shen, T. Chen, J. Li, M. Gillette, R. C. Ransom, M. Ezran, C. A. Johnson, A. B. Castillo, W. J. Shen, F. B. Kraemer, A. A. Smith, J. A. Helms

**Affiliations:** 1 Division of Plastic and Reconstructive Surgery, Department of Surgery, Stanford School of Medicine, Stanford, CA USA; 2 Paris Descartes University - Sorbonne Paris Cité, EA 2496 - Orofacial Pathologies, Imaging and Biotherapies Lab and Dental Medicine Department, Bretonneau Hospital, HUPNVS, AP-HP, Paris, France; 3 Department of Mechanical and Aerospace Engineering, New York University Polytechnic School of Engineering, Brooklyn, NY USA; 4 Division of Endocrinology, Gerontology and Metabolism, Stanford University School of Medicine, Veterans Affairs Palo Alto Health Care System, Palo Alto, CA USA; 5 Present Address: State Key Laboratory of Oral Diseases, West China Hospital of Stomatology, Sichuan University, Chengdu, China; 6 Present Address: Department of Plastic Surgery, University of Pittsburgh Medical Center, Pittsburgh, PA USA

Correction to: *Scientific Reports* 10.1038/s41598-017-14395-9, published online 27 October 2017

The Acknowledgements section in this Article is incorrect.

“The authors would like to thank T. Doyle at Stanford’s Live Imaging Center for his guidance, advice, and generous support for this project. This work was funded in part by a grant from CIRM (TR1-01249) to J.A.H., gift funds to Stanford University and supported by grant from the Philippe Foundation to B.S.”

should read:

“The authors would like to thank T. Doyle at Stanford’s Live Imaging Center for his guidance, advice, and generous support for this project. This work was funded in part by a grant from CIRM (TRAN1-09270) to Ankasa Regenerative Therapeutics, Inc., gift funds to Stanford University and supported by grant from the Philippe Foundation to B.S.”

