# Supplementary material for: WNT-activated bone grafts repair osteonecrotic lesions in aged animals
Source: Sci Rep. 2017 Oct 27;7:14254. doi: 10.1038/s41598-017-14395-9 (PMC5660190; doi:10.1038/s41598-017-14395-9)
Supplement: Supplementary file 1 — Supplementary information [file 41598_2017_14395_MOESM1_ESM.pdf]

# WNT-activated bone grafts repair osteonecrotic lesions in aged animals

Salmon B[1,2], Liu B[1], Shen E[1], Chen T[1,3], Li J[1,3], Gillette M[1], Ransom RC[1], Ezran M[1], Johnson CA[1,4], Castillo AB[5], Shen WJ[6], Kraemer FB[6], Smith AA[1], Helms JA*[1]

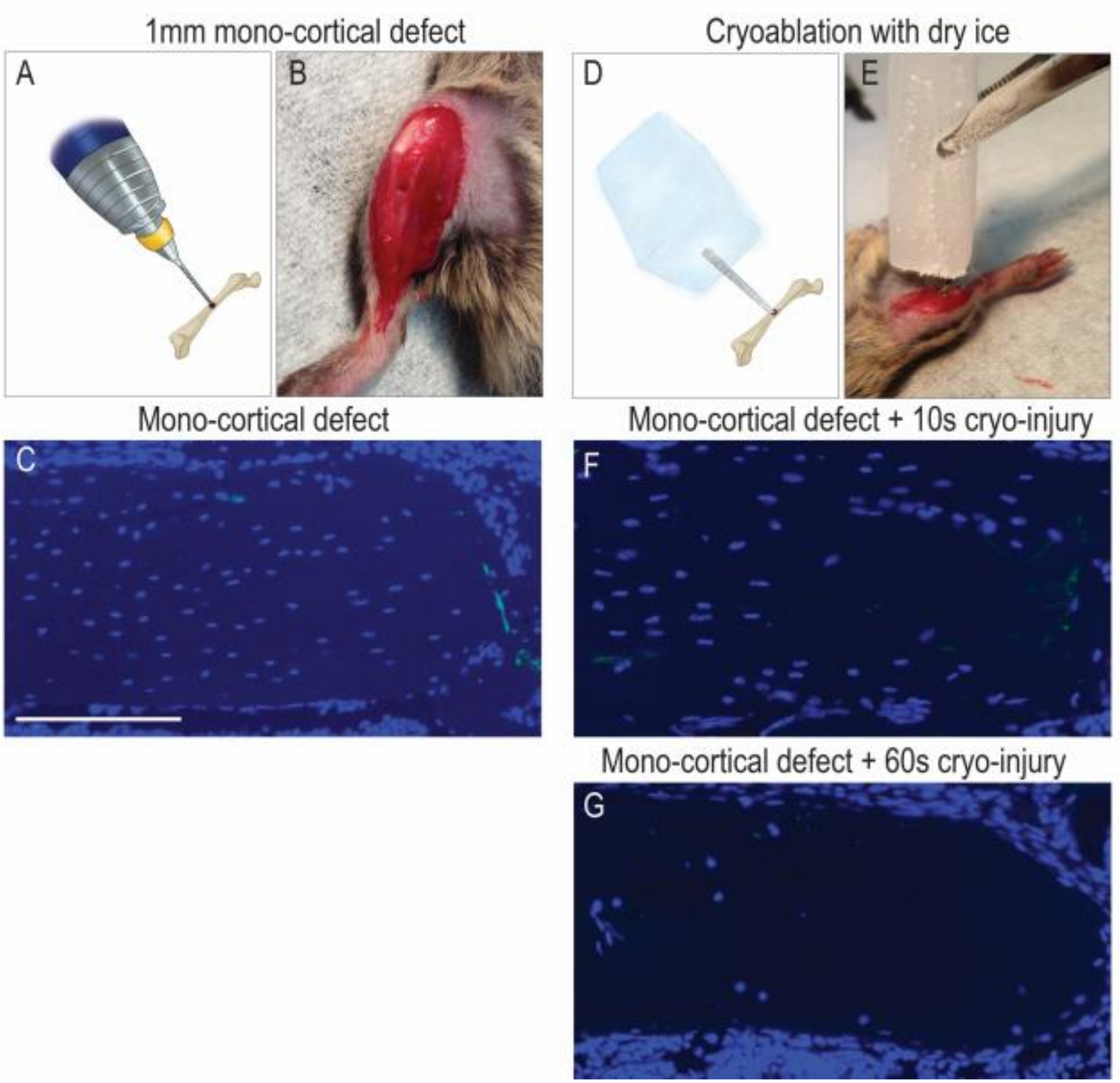


*Supplemental Figure 1. Schematic of the osteonecrotic mouse model and confirmation of cell death by cryoablation.*

(A,B) To expose the bone marrow space, a mono-cortical defect is performed in either the femur or tibia using a calibrated 1.0mm diameter drill. (C) DAPI staining focusing on the cortical edge of the defects, reveals the distribution of viable osteocytes. (D,E) To create necrosis of the bone a 1.0mm diameter drill is used as a cryo-probe to conduct the low-temperature from the dry ice to the defect. DAPI staining shows remaining viable osetocyte nuclei inside the cortical edge after (F) 10sec and (G) 60sec of cryoablation. Scale bars, 100µm; same magnification was applied in panels C,F,G.

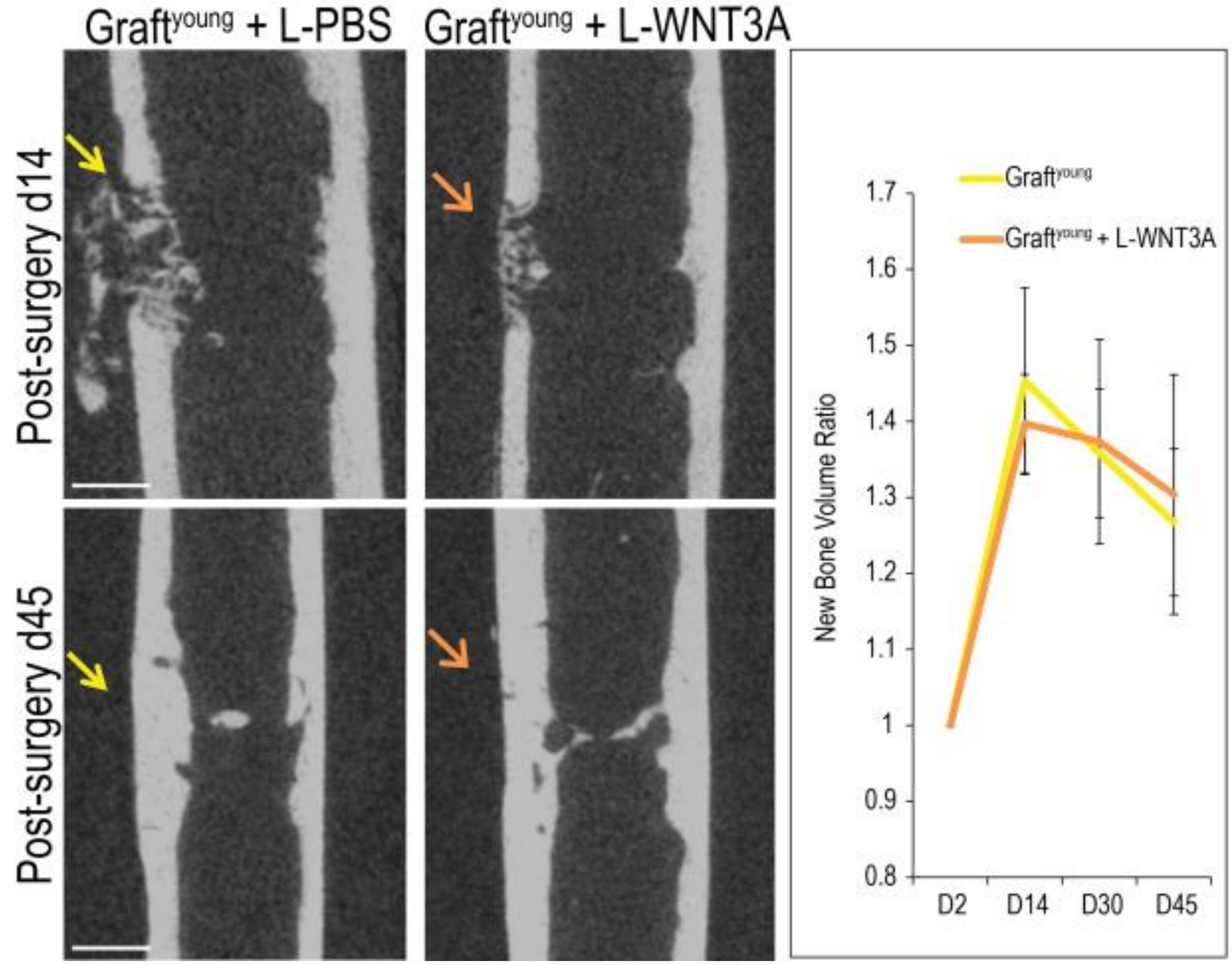


*Supplemental Figure 2. Graft$^{young}$ and graft$^{young}$ + L-WNT3A perform equivalently in healing osteonecrotic defects.*

Micro-CT image quantification from post surgery days 14 and 45 show similar extents of healing between those animals receiving graft$^{young}$ + L-PBS and those treated with graft$^{young}$ + L-WNT3A. Scale bars, 0.5mm; same magnification was applied in all panels.

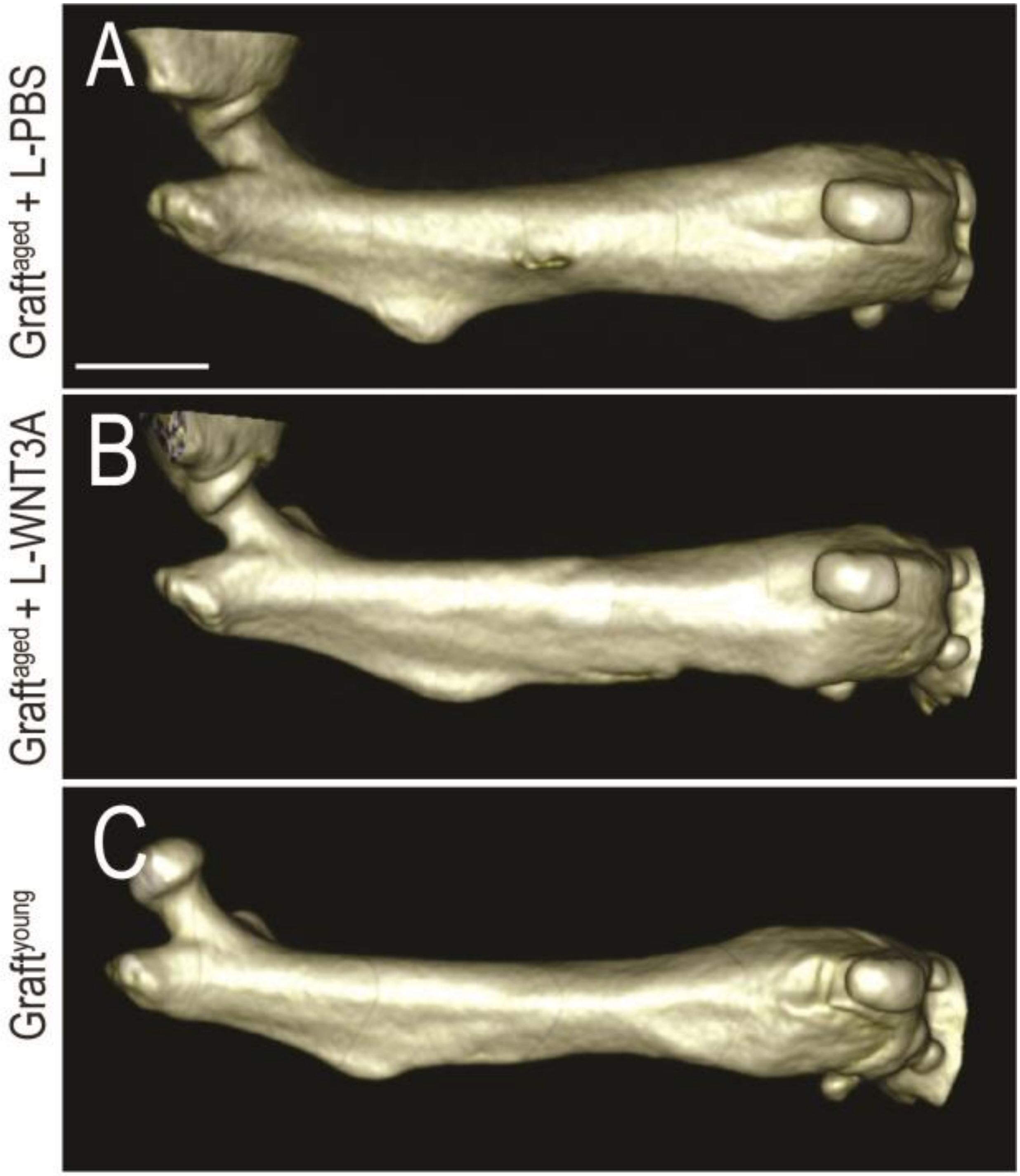


*Supplemental Figure 3. Bone grafting results in healing of osteonecrotic defects.*

On post-surgery day 30 surface rendering showed nearly detectable concavities associated with the osteonecrotic defects, although slight surface irregularities were detected in animals treated with

graft$^{aged}$. No ectopic bone was detected in any treatment group (N≥4 for each condition). (A) graft$^{aged}$ + L-PBS, (B) graft$^{aged}$ + L-WNT3A and (C) graft$^{young}$. Scale bars, 2mm; same magnification was applied in panels A-C.
